# Acoustic vocal measures in women without voice complaints and with normal larynxes

**DOI:** 10.1016/S1808-8694(15)30663-7

**Published:** 2015-10-19

**Authors:** Leila Susana Finger, Carla Aparecida Cielo, Karine Schwarz

**Affiliations:** 1 Master's degree in Human Communication Disorders, UFSM. Clinical speech therapist; 2 Doctorate in Applied Linguistics, PUC-RS. Adjunct professor of the Speech Therapy Department, UFSM-RS; 3 Doctoral student in Neuroscience, UFRGS. Clinical speech therapist. Universidade Federal de Santa Maria

**Keywords:** health evaluation, voice disorders, phonation, voice training, voice

## Abstract

It is important to establish normal voice standards in order to help guide voice professionals.

**Aim:**

to describe acoustic voice measures of adult young women with normal larynxes and without voice complaints.

**Method:**

56 women underwent ENT evaluation and speech screening. The “A” vowel utterance was digitally recorded and analyzed by means of the Praat (Version 4.6.10) software. The data was analyzed by means of descriptive statistics and by the Shapiro-Wilk test with a 5% significance level. The study was cross-section and exploratory.

**Results:**

normal distribution measures were: fundamental frequency; Jitter (local); Jitter (local, absolute); Jitter (ppq5); Jitter (ddp). The Jitter (rap), all the Shimmer, the noise/harmonic ratio (NHR) and the harmonic/noise ratio (HNR) values did not follow a normal distribution.

**Conclusion:**

It seems that the measures which followed the normal distribution can be used as base-normal values for the interpretation of acoustic voice analysis of those women with and without laryngeal disorders. All the values with and without normal distribution showed results similar to the ones present in the national and international literature.

## INTRODUCTION

Computerized acoustic voice analysis is one of the major advances in the study of voice, increasing the accuracy of diagnosis in this area. It is a procedure that, together with developments in the understanding of voice physiology and science,^1^ has provided clinicians with abundant objective and quantified data to better understand phonation; human voice is thus close to being fully described.^2^,^3^

Acoustic voice analysis provides normative or fundamental data for different voice realities. A significant amount of information gained in acoustic analysis is still little known, and its exploration has not generally been stimulated. Among acoustic measures that voice laboratories offer, those with clinical applications are: the fundamental frequency, voice intensity, noise measures, and frequency and intensity perturbation measures.^4^

Normal standards are important for guiding voice professionals, since normal voice varies widely, given that it is a personal feature and no voice is perfectly equal to another.^2^,^5^

Normative or normal standards are needed based on the extraction and quantification of precisely defined voice signal standards^3^ to guide voice care professionals, particularly because of a paucity of studies with acoustic measures of normal voice in young female adults.

Acoustic analysis software is able to trace sound wave forms by processing signals and applying algorithms. Thus, analyses of the fundamental frequency, perturbation measures such as jitter and shimmer, and measures of noise make it possible to describe the human voice almost completely.^2^

Because of technological and scientific advances in the area of voice, there are a variety of computerized voice assessment software in the market; each one has its advantages and disadvantages.^4^ In the Brazilian literature, there are reports about a number of acoustic analysis software, such as the Praat, the Multi-Dimensional Voice Program (MDVP, Kay Elemetrics), he Doctor Speech Sciences (Tiger Elemetrics), the CSL 4300 (Kay Elemetrics), and the Analise de Voz (DSP Instrumentos Ltda).^1^, ^2^, ^3^, ^4^, ^5^, ^6^

The lack of papers in the Brazilian literature on the Praat software,6 which is free, easy to use, and provides website discussion groups among users and the software creators for clarifying doubts, together with an increasing number of published international studies,^7^, ^8^, ^9^, ^10^, ^11^, ^12^ led us to undertake this study.

The authors of Praat^13^ propose an analogy between their analysis values and the normal values based on the Multi-Dimensional Voice Program (MDVP, Kay Elemetrics), a trend that was confirmed in a later study.^8^

The purpose of this study was to describe acoustic voice measurements in female subjects with normal larynxes and no voice complaints. A second purpose was to demonstrate that the Praat software and the Multi-Dimensional Voice Program (MDVP, Kay Elemetrics) are similar, given that the Praat software is more easily accessible. An additional purpose was to compare the results of this study with the findings in other studies that applied different acoustic voice analysis software, such as the Doctor Speech Sciences (Tiger Elemetrics), the CSL 4300 (Kay Elemetrics), and the Analise de Voz (DSP Instrumentos Ltda).

## MATERIAL AND METHOD

### Study design and ethical aspects

This was a quantitative and qualitative cross-sectional and exploratory study based on field data. The Research Ethics Committee of the institution of origin approved this study (protocol number 024/2006). Data gathering started after all subjects read and signed a free informed consent form based on the Resolution 196/96 of the National Research Ethics Committee (CONEP).

### Research subjects

In order, inclusion criteria were: female sex, since there are more studies involving female patients^14^,^15^ and more ease in obtaining volunteers; age from 18 to 40 years, since until this age the phonation apparatus has not been affected by age-related hormonal and structural changes,^16^ or voice changes that in women occur from 12 to 14 years;^17^ compliance with the free informed consent form.

Exclusion criteria were: a history of neurological, psychiatric, endocrinological,^18^,^19^,^20^ or gastric diseases^10^,^21^ that might alter voice performance or understanding of orders during the evaluation;^19^ voice complaints such as hoarseness, voice fatigue, voice failure or irritated throat, since these symptoms suggest organic or behavioral alterations of voice^18^,^19^ that might affect study results; laryngeal conditions, since these disorders could compromise the results;^18^,^19^ hormonal alterations due to pregnancy or the menstrual and premenstrual period,^22^ which were established during the clinical history taking; presenting a common cold or respiratory allergies - since these conditions may cause vocal fold edema - or other diseases that could limit voice production on the evaluation day,^20^,^23^ use of alcoholic beverages^18^,^19^ and smoking,^10^,^18^ since alcohol and tobacco may harm the larynx and cause organic voice conditions; having undergone prior speech therapy and/or otorhinolaryngological treatment, to discard subjects with voice affections (even if treated) or voice conditioning by training with voice techniques;^14^,^15^ auditory alterations, since these may alter self-monitoring of voice and compromise voice quality;^18^,^19^ stomatognathic system alterations that might interfere with articulation of speech and compromise voice;^18^ singing in choirs, to avoid subjects with trained voices.

Thus, acoustic measurements were made of the voices of 56 adult female subjects with no voice complaints and with normal larynxes, aged from 18 to 38 years (mean– 23 years).

### Data gathering and sampling procedures

After signing the free informed consent form, subjects answered a questionnaire, and underwent an otorhinolaryngological assessment and speech therapy screening, which consisted of an orofacial myofunctional evaluation and an auditory assessment. Fifty-six subjects were selected for evaluation based on the inclusion criteria.

Auditory screening consisted of air conduction pure tone scans at 500, 1000, 2000 and 4000 Hz (25 dB).^24^

Volunteers with altered evaluations were excluded and referred for a more complete assessment. Data gathering was started with eligible subjects. Initially, the sustained vowel /a/ was measured; for this subjects stood with arms extended along the body. A microphone coupled to a digital recorder (Creative Labs, MuVo Tx FM model) was positioned 90° to the subject's mouth at a distance of 4 cm.^8^,^9^,^18^,^25^ The subject was asked to issue the sustained vowel at a habitual frequency and intensity following a deep breath, issuing the sound to achieve maximum phonation time without using the expiratory reserve air.

The initial 3.5 seconds of vowel /a/ emission was extracted for the voice acoustic analysis; the beginning of emission was excluded to avoid interference from voice attach on data analysis.^4^,^11^,^26^

Praat software (version 4.6.10) measures,^13^ used successfully in many studies of voice,^7^, ^8^, ^9^, ^10^, ^11^, ^12^ were: the fundamental frequency (f0); minimum frequency; maximum frequency; jitter (local); jitter (local, absolute); jitter (rap); jitter (ppq5); jitter (ddp); shimmer (local); shimmer (local, dB); shimmer (apq3); shimmer (apq5); shimmer (apq11); shimmer (ddp); noise-harmonic ratio (NHR); and harmonic-noise ratio (HNR). These measures comprise all of the possible measures provided by this software; their importance for analysis resides in the fact that they provide information about voice signal aperiodicity, stability, noise, and frequency levels.

Female f0 values proposed by Behlau, Tosi & Pontes (150 to 250 Hz)^27^ were applied in this study; they comprise the mean and/or normal frequency range found in many subsequent studies.^2^,^3^,^5^,^14^,^15^,^26^,^28^,^29^ (Table 1)

**Table 1:** Normal values for the female f0.

Study	Normal range (Hz)	Mean (Hz)
Behlau, Tosi and Pontes (1985)	150 a 250	–
Araújo et al. (2002)	–	215,42
Andrade (2003)	172,44 a 286,48	209,61
Guimarães e Abberton (2005)	199 a 215	–
Santos (2005)	–	208,9
Siqueira and Moraes (2005)	208,99 a 220,57	214,78
Brum (2006)	190 a 225	–
Felippe, Grillo & Grechi (2006)	–	207
Schwarz (2006)	168,55 a 246,62	203,49

Boersma and Weenick13 have proposed an analogy with values considered as normal in the Multi-Dimensional Voice Program (MDVP, Kay Elemetrics) for some of the remaining measures in the Praat software. (Table 2)

**Table 2:** Analogy among normal values - Praat × MDVP.

Praat measure	MDVP equivalent measure	MDVP normal	Standard deviation	Tresh
MDVP				
Jitter local (%)	Jitt	0,633	0,351	<1,040
Jitter local, absolute (ms)	Jitta	26,927	16,654	< 83,200
Jitter rap (%)	Jitter rap	0,378	0,214	< 0,680
Jitter ppq5 (%)	Jitter (PPQ)	0,366	0,205	< 0,840
Jitter ddp (%)	Praat Original	–	–	–
Shimmer local (%)	Shim	1,997	0,791	< 3,810
Shimmer local dB (dB)	ShdB	0,176	0,071	< 0,350
Shimmer apq3 (%)	Praat Original	–	–	–
Shimmer apq5 (%)	Praat Original	–	–	–
Shimmer apq11 (%)	APQ	1,997	0,527	< 3,070
Shimmer dda (%)	Praat Original	–	–	–
NHR	NHR	0,112	0,009	< 0,190
HNR (dB)	Praat Original	–	–	–

**Figure 1:**
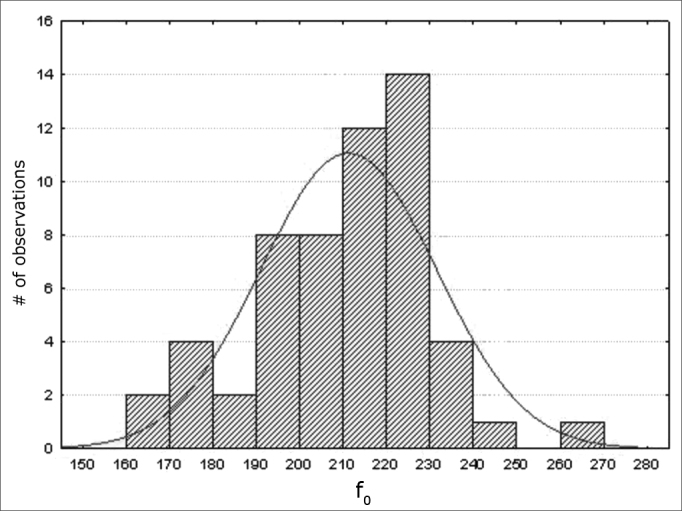
Normal distribution of the fundamental frequency - Histogram

**Figure 2:**
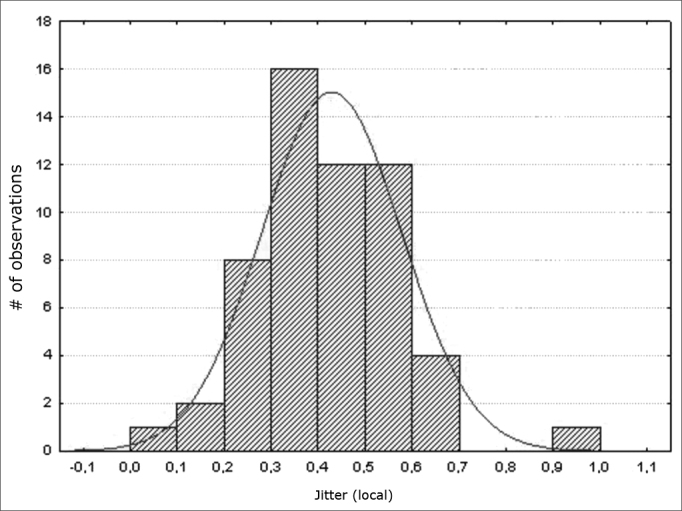
Normal distribution of jitter (local) - Histogram

**Figure 3:**
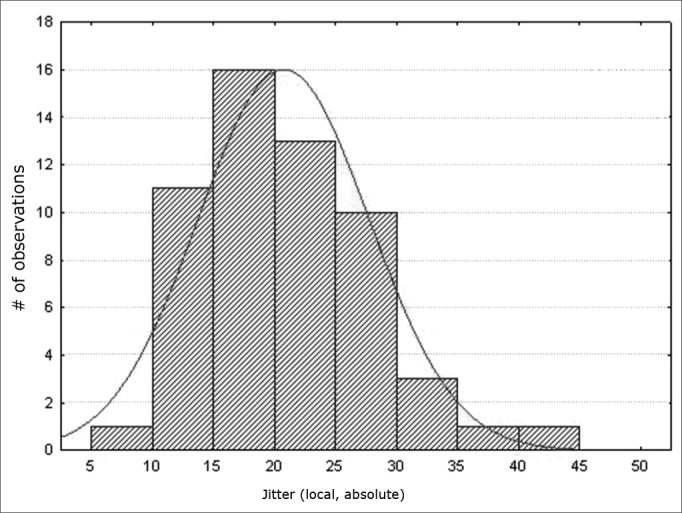
Normal distribution of jitter (local, absolute) - Histogram

**Figure 4:**
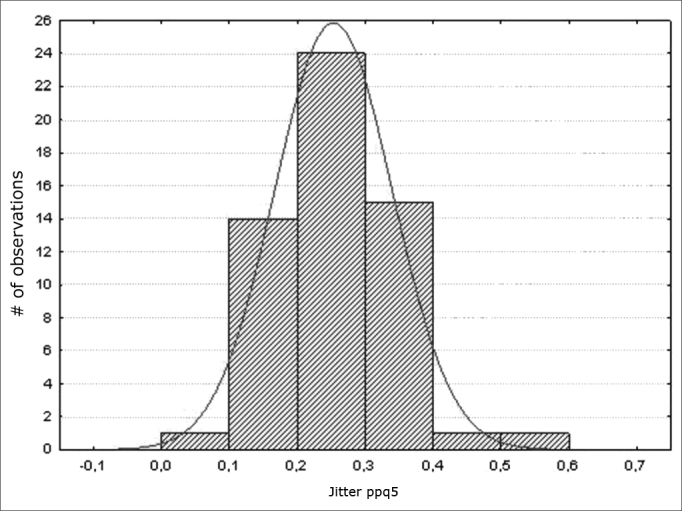
Normal distribution of jitter (ppq5) - Histogram

**Figure 5:**
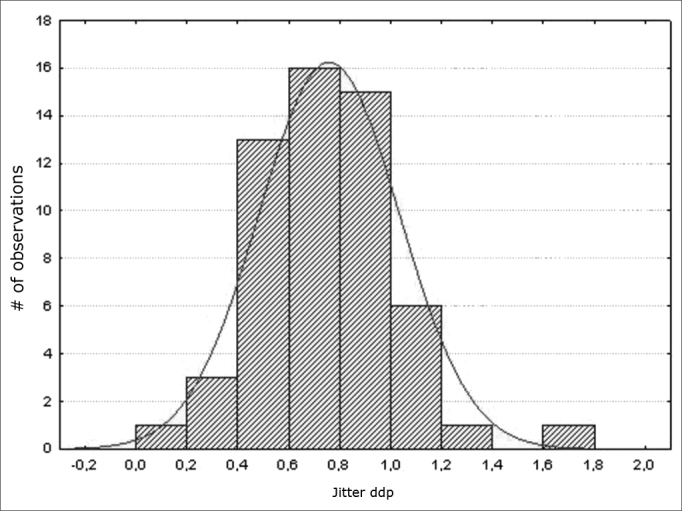
Normal distribution of jitter (ddp) - Histogram

Data were assessed statistically by applying descriptive statistics. The Shapiro-Wilk was applied for assessing the normality of results; the significance level was 5% (p> 0.05); this yielded a results distribution curve (Figure 1, Figure 2, Figure 3, Figure 4, Figure 5).

## RESULTS

Variables f0; jitter (local); jitter (local, absolute); jitter (ppq5); jitter (ddp) had a normal distribution (a normality baseline standard), as shown in Figure 1, Figure 2, Figure 3, Figure 4, Figure 5.

## DISCUSSION

Because of recent significant technological developments, acoustic analysis is more disseminated among speech therapists; however, different software and their various extraction measures still hinder comparisons of data from different studies.^3^,^28^

Given the paucity in the Brazilian literature of published papers applying the Praat^6^ software, which was used in this study for the acoustic analysis of voice, we decided to conceptually discuss the results based on existing research on this theme, even with different software.

Many published studies on voice conditions in the international literature have demonstrated the functionality of the Praat software for differentiating pathological from normal voice.10 Since it is free and easily used software, it has been applied in voice research worldwide.^7^, ^8^, ^9^, ^10^, ^11^, ^12^ There is an online discussion group on its website to foster debates and clearing of doubts among users and software creators.

It is important to establish normal standards to guide speech therapists on what is normal voice; not only do measures vary when measured by different software, but also there is a wide range of normal voices. This fact is possibly due to individual differences, since voice is a personal feature, and no voice is perfectly equal to any other.^2^,^3^ Production of voice also depends on adequately functioning respiratory, cardiovascular, muscular-skeletal, neurological and psychosocial systems in individuals.^20^

The f0 is one of the most frequently used measures by clinicians to characterize human voice; it yields cues about age, sex and individual height.^3^,^5^ The f0 is the number of glottic cycles per second, and is related with mechanisms such as vocal fold length, mass and strain. Thus, lengthening the vocal folds will cause the glottic cycles to occur faster, yielding more acute resulting frequencies.^4^ Variations of this measure also result from other factors, such as different speech tasks (sustained vowels, reading, conversation, singing) different languages and dialects, smoking, stress, dysphonia and analysis forms.^5^

Measures of the f0 in this study (Table 3; Fig. 1) had a normal distribution, with values markedly concentrated around 210 and 220 Hz; this variation range and mean values are similar to those proposed by Behlau, Tosi & Pontes^27^ (150 to 250 Hz), which is considered the reference female f0 in Brazil.

**Table 3:** Results of acoustic voice analysis.

Variable	< value	> value	Mean	SD	SW-W	p	Normal	Tresh
f0 (Hz)*	164,60	268,94	210,92	20,17	0,9703	0,1812	–	–
Frequência								
mínima (Hz)	95,53	259,33	199,10	30,49	0,08416	0,0003	–	–
Frequência								
máxima (Hz)	145,17	273,86	214,99	22,20	0,957812	0,0481	–	–
Jitter local (%)*	0,032	0,972	0,426	0,148	0,959001	0,0546	0,633	<1,040
Jitter local,								
absoluto (ms)*	8,568	41,85	20,647	6,978	0,963804	0,0910	26,927	< 83,200
Jitter rap (%)	0,106	0,585	0,256	0,088	0,946248	0,0145	0,378	< 0,680
Jitter ppq5 (%)*	0,012	0,553	0,251	0,086	0,963	0,0831	0,366	< 0,840
Jitter ddp (%)*	0,062	1,754	0,753	0,275	0,9644	0,0965	–	–
Shimmer								
local (%)	1,393	4,861	2,964	2,199	0,903555	0,0003	1,997	< 3,810
Shimmer								
local dB (dB)	0,122	0,692	0,268	0,197	0,8961	0,0002	0,176	< 0,350
Shimmer								
apq3 (%)	0,760	2,763	1,789	1,068	0,922022	0,0014	–	–
Shimmer								
apq5 (%)	0,890	4,977	2,109	1,488	0,906422	0,0004	–	–
Shimmer								
apq11 (%)	1,060	5,981	2,753	2,170	0,897392	0,0002	1,997	< 3,070
Shimmer								
dda (%)	2,281	9,089	5,063	3,210	0,92163	0,0014	–	–
NHR	0,002	0,996	0,040	0,135	0,231068	0,0001	0,112	< 0,190
HNR dB	12,64	26,51	19,332	3,688	0,944217	0,0118	–	–

* Variables with a normal distribution - Shapiro-Wilk Test

These results are also similar to the mean f0 value of 203.49 Hz in the 168.55 to 246.62 Hz range found by Schwarz15 and the f0 variation of 190 to 225 Hz found by Brum^14^ in studies of female subjects with no voice alterations and normal larynxes; theses authors applied the Multi-Dimensional Voice Program (MDVP, Kay Elemetrics).

This trend is also seen in Andrade's^26^ study of 77 female subjects, in which the Analise de Voz software, version 2.0 (DSP Instrumentos Ltda) showed a mean f0 value of 209.61 Hz, ranging from 172.44 to 286.48 Hz. Santos^3^ analyzed the voices of 38 young female subjects to compare acoustic voice measures between young adults and elderly subjects, using a new version of the Analise de Voz software - which Andrade^26^ had used - and found a mean f0 value of 208.90 Hz. Felippe, Grillo & Grechi 29 analyzed the voices of 20 female subjects using the CSL– 4300 program (Kay Elemetrics) and found a mean f0 value of 207 Hz.

Araujo et al.'s^2^ study of 40 female subjects, also using the Analise da Voz software, found a mean f0 of 215.42 Hz when issuing the vowel /a/. Siqueira and Moraes^28^ (2005) studied 50 female voices using the Doctor Speech Sciences software (version 4.0, Tiger Eletronics) and found a mean f0 value of 214.78 Hz, which is similar to the abovementioned results and with those of our paper (Table 3; Fig. 1).

Guimarães & Abberton5 found f0 measures ranging from 199 to 215 Hz using electroglottography applied upon the sustained emission of the vowel /a/ in 82 Portuguese women (speakers of European Portuguese). This range is similar to our own results (Table 3, Fig. 1) and to the other Brazilian studies mentioned above.

Cycle-to-cycle perturbation measures assess acoustic signal variations; they relate to how much a specific glottic vibration period is different from the ensuing period with relation to frequency (jitter) and intensity (shimmer). It is important to note that the results of jitter e shimmer measures depend on the method applied in each software, and may vary with age, sex and the vowel that is used; these measures are not yet standardized.^3^,^4^,^26^

Vieira & Rosa^25^ have suggested that acoustic measures based on three or more glottic cycles are more reliable. These authors argue that such an approach reduces cycle-to-cycle perturbations and avoids period defining errors, thus increasing the reliability of measures.

Jitter, which is voice frequency cycle-to-cycle perturbation,^4^,^10^ is an objective and reproducible measures that evaluates minor glottic pulse irregularities; it reflects hoarseness^20^ or voice noise.

Measures of local jitter (Fig. 2), which is the mean absolute difference between consecutive periods, divided by the mean period; of local absolute jitter (Fig. 3), which consists of the mean absolute difference between consecutive periods; of jitter (ppq5) (Fig. 4), which is the mean absolute difference between a period and the mean of that period and the closest four neighboring periods, divided by the mean period; and of jitter (ddp) (Fig. 5), the mean absolute difference among consecutive differences between consecutive period, divided by the mean period;^13^ had a normal distribution in this study (Table 3).

Schwarz's^19^ study using MDVP (Kay Elemetrics), with analogous results to those with the Praat software,^9^,^13^ reported a mean jitter value of (ppq) 0.56%, ranging from 0.25 to 1%. This results is similar to that of Brum,^14^ who also used the MDVP for analysis, in which the range was from 0.33 to 1.5%. Our results are similar to those in these papers (Table 3; Fig. 4), in which values were strongly concentrated between 0.1 and 0.4%.

Oguz et al.^11^ found mean jitter (local) values of 0.3%; mean jitter (local, absolute) of 1.227ms; and jitter (ppq5) of 0.17%; this author applied the Praat software in a study of Turkish women with no voice alterations.

These values are within our range (Table 3; Fig. 2; Fig. 3; Fig. 4; Fig. 5). This trend was also seen in another study also using the Praat software to assess male and female subjects in Turkey, in which the mean values were: jitter (local) – 0.29%; jitter (local, absolute) – 17.172ms; and jitter (ppq5) – 0.17%.

In our study, jitter (ddp) measures were concentrated between 0.4 and 1% (Fig. 5); however, there were no reports of extractions of this measure in female voices with no voice alterations, which would have made it possible to discuss the results of this study.

Among jitter measures, only jitter (rap), which consists of the mean absolute difference between a period and its mean plus that of its two neighboring values divided by the mean period,^13^ did not have a normal distribution in this study (Table 3). Oguz et al.^11^ found a mean jitter (rap) measure of 0.17%. These mean values are within the range found in our study (Table 3), which is similar to the jitter (rap) value of 0.16% encountered by Oguz et al.^10^.

Siqueira and Moraes^28^ measured a jitter value of 0.36% (ranging from 0.33% to 0.40%), using the Doctor Speech Sciences (version 4.0) software; these values were within the normal range suggested by the program (values equal to or lower than 0.5%). These authors added that the relative jitter is used in Doctor Speech, which is expressed as a percentage relative to the mean f0. There were, however, no comments about whether these values could be compared to those gathered in the Praat software.

Fellipe, Grillo and Grechi^29^ found jitter values of 0.624% in an acoustic voice analysis of 20 female subjects with no voice symptoms or health issues; these authors applied the CSL– 4300 software (Kay-Elemetrics). There are different methods for extracting jitter, such as absolute jitter, relative jitter, RAP (relative average perturbation), PPQ (pitch perturbation quotient), and others.^4^ However, the authors did not specify the extraction method for these values, which makes comparisons with our results difficult (Table 3), since many jitter measures in the Praat software - which are compatible with those of the MDVP program (Kay Elemetrics) - also use % as the unit.

Shimmer measures in our study were not distributed normally (Table 3). These measures reflect cycle-to-cycle amplitude perturbations; their increase is related with a decreased or inconsistent vocal fold contact coefficient. Furthermore, these measures may also be related with voice soprosity10 or noise in general.

Shimmer (local, dB) is the absolute mean (log10) of amplitude differences between consecutive periods multiplied by 20; shimmer (local) is the absolute mean difference between amplitudes of consecutive periods divided by the general mean amplitude; shimmer (apq3) is the mean absolute difference between the amplitude of a period and the mean amplitudes of its neighbors divided by the mean amplitude; shimmer (apq5) is the mean absolute difference between the amplitude of a period and the mean of its amplitude and that of its closest four neighbors divided by the general mean amplitude; shimmer (apq11) is the mean absolute difference between the amplitude of a period and the mean of its amplitude and that of its ten closest neighbors divided by the general mean amplitude; and shimmer (dda) is the mean absolute difference between consecutive amplitude differences of consecutive periods.

The mean shimmer (APQ) measure of 2.46% (ranging from 1.68 to 5.61%) found by Schwarz^15^ is similar to our results (Table 3) and those of Brum,^14^ in which shimmer (APQ) ranged from 1.29 to 2.04%.

Oguz et al.^11^ found mean shimmer (local) values of 4.42 %; shimmer (local, dB) of 0.4 dB; shimmer (apq3) of 2.37%; and shimmer (apq5) of 2.98%. Shimmer (local) and shimmer (local, dB) results were higher than the maximum normal values suggested by the analysis program. Oguz et al.'s^11^ shimmer measures were much higher than our results, except for shimmer (apq11) (Table 3).

This trend in mean shimmer values is also found in another study by Oguz et al.,10 in which the results were: shimmer (local) – 4.54%; shimmer (local, dB) – 0.4 dB; shimmer (apq3) – 2.59%; and shimmer (apq5) – 2.70%. A shimmer (apq11) value of 0.157%, similar to shimmer (APQ) in Schwarz's15 and Brum's14 study, and also our own results (Table 3), was within the normal range suggested by the analysis program.

Values out of the normal range suggested by analysis programs, the high standard deviation values found in the Turkish studies,^10^,^11^ the high standard deviation values found in our study (Table 3), and the significant shimmer variation range found by Schwarz,^19^ comprise results that are similar to those of Behlau et al.;^18^ these authors consider shimmer a measure that requires further investigation to provide more conclusive results, given its variability.

NHR (noise/harmonic ratio) and HNR (harmonic/noise ratio) measures are inversely proportional values that assess the presence of noise in a voice signal; they are directly related with voice quality. A lower NHR and a higher HNR indicate superior voice quality. They reflect a general assessment of noise in a given signal; they are not specific for any cycle, and include amplitude and frequency perturbations. These measures establish a general perception of noise and hoarseness in a voice signal.^11^

HNR measures in our study (Table 3) were not distributed normally; the variation range is around a mean value of 24.24 dB, which was measured by Siqueira & Moraes^28^ using the Doctor Speech software, version 4.0 (Tiger Elemetrics).

NHR values in our study (Table 3) were also not distributed normally, but are similar to Brum's^14^ results (ranging from 0.03 to 0.14) and Schwarz's^15^ results (mean – 0.14; ranging from 0.09 to 0.17). These values and those in our study (Table 3) are also similar to those of Oguz et al.10 (0.157) and Oguz et al.^11^ (0.016).

## CONCLUSION

We were able to conclude that, in acoustic measures of voice in female subjects with normal larynxes and no voice complaints, f0, jitter (local), jitter (local-absolute), jitter (ppq5), and jitter (ddp) measures were distributed normally, and may be used as normal baseline values for interpreting the results of acoustic analyses of the female voice with or with no laryngeal disease. Shimmer, NHR and HNR measures did not have a normal distribution.

These measures, with a normal distribution or otherwise, were similar to other published results in the Brazilian and international literature. Differences among acoustic voice analysis software were minimal, as were different analyses using the same program; thus, contrary to what might be expected, different populations may be assessed using different software to provide similar results on the same measures or their equivalents.
